# Eco-Sustainable Magnesium Oxychloride Cement Pastes Containing Waste Ammonia Soda Residue and Fly Ash

**DOI:** 10.3390/ma15175941

**Published:** 2022-08-28

**Authors:** Qing Wang, Wenjie Huang, Yuhang Liang, Congbo Li, Mianheng Lai, Jing Sun

**Affiliations:** School of Civil Engineering, Guangzhou University, Guangzhou 510006, China

**Keywords:** fly ash, magnesium oxychloride cement, ammonia soda residue, expansion, waste recycling

## Abstract

Magnesium oxychloride cement (MOC), a type of special construction material, has drawn much research attention in solid waste utilization and environmental protection due to its eco-friendly production. Ammonia soda residue (ASR), a by-product generated from sodium carbonate manufacturing, is one of the industrial wastes that can be recycled in MOC systems. However, ASR exhibits adverse effects on the fresh performance and volume stability of MOC pastes. This paper aims at improving the properties of ASR-MOC by introducing fly ash (FA), solid waste from the power industry. Firstly, the roles of FA in MOC pastes are evaluated and analyzed. Then, three substitution ratios of FA (33.3%, 50% and 66.7% in weight) for ASR are designed for MOC pastes with 10% to 40% industrial wastes. Flowability, setting, strength and expansion of all mixtures were experimentally studied. Furthermore, X-ray diffraction (XRD) and scanning electron microscope (SEM) approaches were adopted to illustrate the microstructure changes. Results show that by adding different amounts of FA, the inferior flowability of MOC caused by ASR can be improved by 6–23%, the setting process can be prolonged by 30–55% and the expansion ratio can be reduced by 14–66%. The intensity of characteristic peaks of 5-phase and Mg(OH)_2_, together with the degrees of crystallization in XRD curves, well explain the strength variation and volume stability of ASR-MOC pastes. According to the regulation of relative specification, up to 20% of solid wastes in weight (10% FA + 10% ASR) can be consumed, contributing greatly to the greener sustainable development of construction materials.

## 1. Introduction

Ammonia soda residue (ASR) is a type of by-product generated in the Solvay process of sodium carbonate manufacture [1,2]. The effluent of ASR produced in the distiller tower is characterized by high pH, large amounts of dissolved solids and abundant ammonia [3]. Typically, ASR contains high content of calcium chloride (CaCl_2_) and sodium chloride (NaCl) because of the inefficient utilization of chloride (<5%). Traditionally, the sewage of ASR is directly discharged into adjacent waterways, causing serious water pollution and soil contamination issues. Due to the large output of solid waste (up to 300 kg per ton of soda ash production), these environmental problems are aggravated, especially in the areas near ammonia-soda factories [4]. Thus, appreciable utilization and recycling have become critical technical points for ASR disposal. Previously, researchers made a great effort in utilizing solid ASR to produce building materials such as cement, geopolymers and concrete, ascribed to its potential hydraulicity and similar physiochemical properties [5,6,7]. Nevertheless, recycled ASR contains a large amount of chloride ions (Cl^−^ > 15%) and magnesium ions (Mg^2+^), harmful to its durability. Therefore, the idea of reusing solid ASR powder in magnesium oxychloride cement (MOC) systems was proposed to mitigate the defects of high Cl^−^ and Mg^2+^ concentration [8]. MOC, produced from magnesium oxide and magnesium chloride mainly, is commonly used in various building materials, such as fire-resistant panels, tiles, decorative components, ventilation pipes, etc. [9,10]. Compared with traditional cement, MOC has the advantages of ultra-high strength, wear and fire resistance. The disadvantage of poor water resistance cannot be ignored, which will decompose the microstructure, resulting in a significant reduction in strength. Nevertheless, the appropriate environment and ultra-high strength of MOC make the reutilization of ASR become feasible and comparatively more effective.

However, previous studies also detected noticeable distinctions of ASR-MOC, mainly in two aspects: acceleration in the setting process and deterioration in volume stability [8]. When 40 wt. % ASR was incorporated, the setting process was shortened to approximately half that of control MOC paste, significantly affecting the workability and operability of fresh MOC mixtures. The expansion ratio was evaluated as 0.244% for MOC paste without ASR, but 0.288%, 0.571%, 0.973% and 1.331% when 10%, 20%, 30% and 40% ASR were added by weight. Herein, the volume expansion of MOC pastes with 40 wt. % ASR is almost 6 times that of the control group, indicating a relatively unstable microstructure in the composite. Active MgO reaction, gypsum hardening induced by volume expansion (0.05–0.15%) and higher internal porosity are the main causes of unstable deformation. To further facilitate the recycling of solid waste previously mentioned in MOC products, the deformation shall be reduced according to the specifications of the corresponding product standard [11]. Therefore, the improvement of flowability and volume stability of MOC with ASR is quite essential.

It is commonly believed that fly ash (FA) particles can help increase the flowability of cementitious material thanks to their lubricating effect on the basis of spherical morphologies [12,13,14,15]. Microstructures of FA-incorporated cementitious composite tend to be denser, which can be attributed to the filling effect of vitreous particles among hydration products [16,17]. Other researchers found that FA can mitigate the free deformation of cement paste [18], high-performance concrete [19] or self-compacting concrete, especially for long-term properties [20]. Herein, the reduction ratio was reported as 14.17%, 17.88% and 21.08% for high-performance concrete incorporated with 33.3%, 40% and 50% FA in weight after curing for 125 days [19]. Thus, the improvement of FA in controlling the volume stability of traditional concrete is undisputed through an inspection of the literature. However, whether such enhancement can also exist in magnesium oxychloride cement systems, especially when incorporated with ammonia-soda residue, is still unknown.

These potential improvements brought by FA can extend the application for ASR-MOC. Firstly, in projects requiring longer setting time, such as subgrade construction, the improved MOC product can perform well. Secondly, it increases the possibility to prepare high-performance concrete with high workability, high strength (>60 MPa) and low deformation [21]. Thirdly, when applied in repair mortar, MOC with a low expansive ratio can reduce the risk of cracking. Therefore, the present study focuses on the influence of vitreous FA particles on the performance of MOC pastes containing ASR, including the setting process, strength development and free deformation. Meanwhile, X-ray diffraction analyses and scanning electron microscope observations were conducted to further study the microstructure of MOC pastes intensively. Then, mixtures of MOC pastes with both ASR and FA were designed and prepared accordingly, aiming at improving the performance of ASR-MOC and extending its application.

## 2. Role of Fly Ash in MOC Pastes

### 2.1. Characteristics of FA

**Figure 1:**
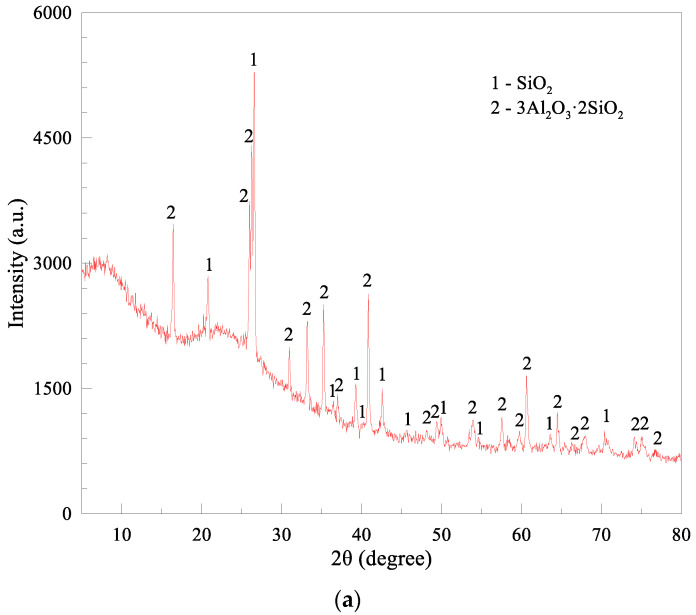

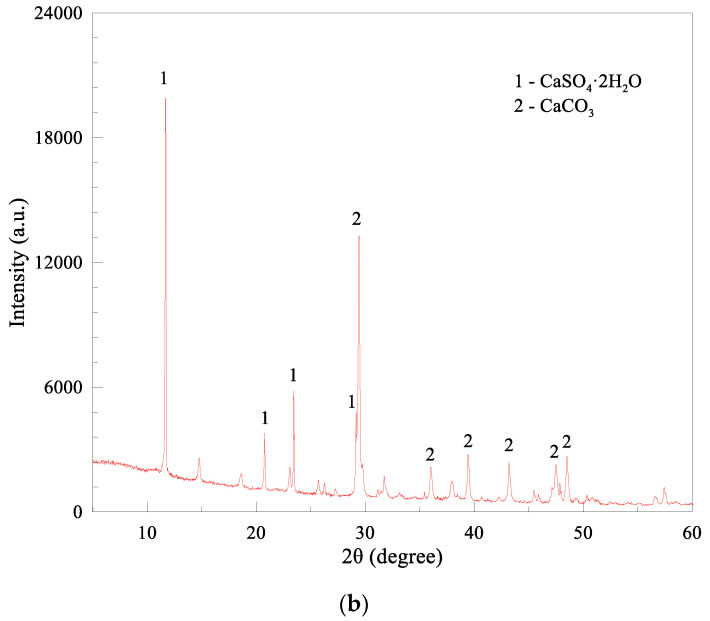
X-ray diffraction curves of solid wastes: (**a**) FA and (**b**) ASR.

**Figure 2:**
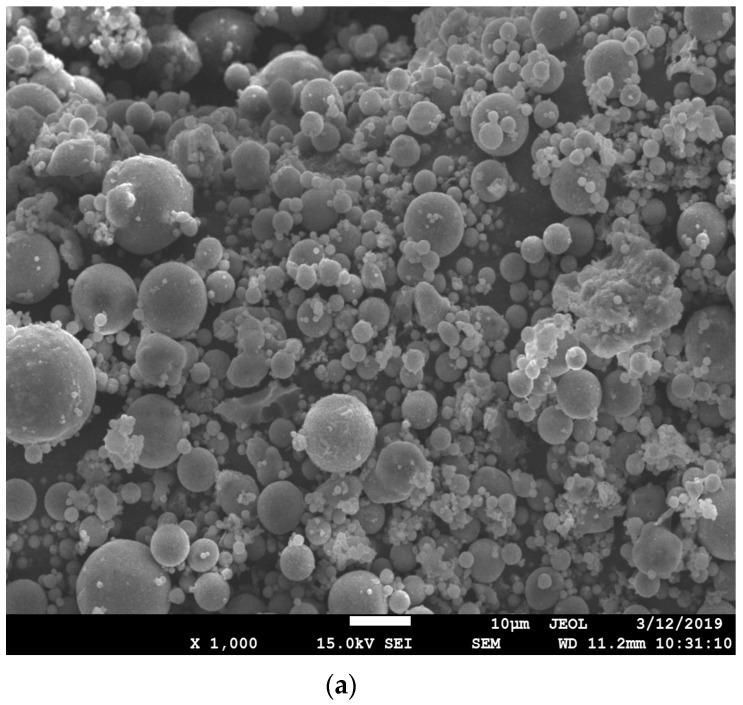

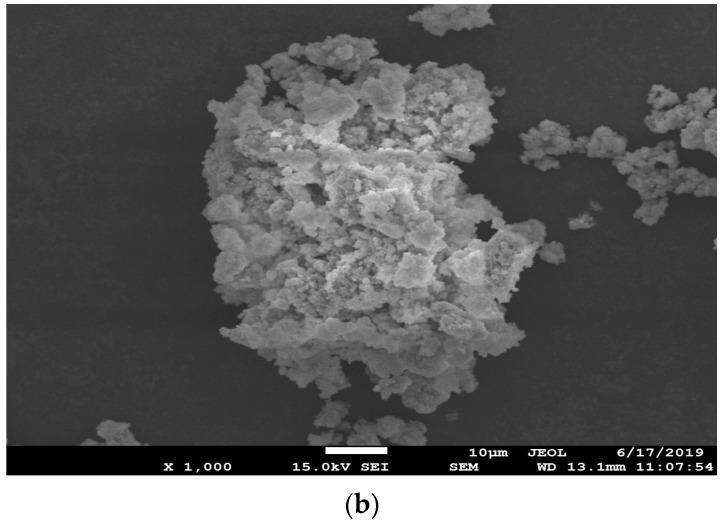
Micro morphologies of solid wastes (**a**) FA and (**b**) ASR.

**Figure 3:**
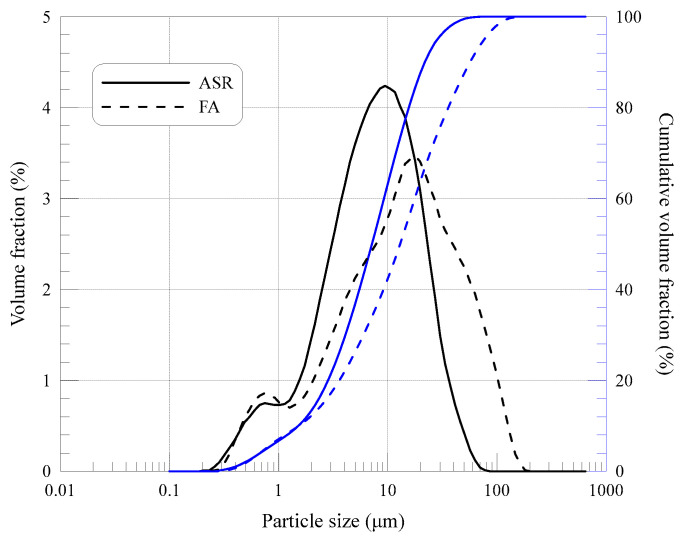
Particle size distribution of ASR and FA.

FA particles were recycled from a local power plant, mainly composed of silicon dioxide and aluminum oxide, in addition to ASR gypsum and calcium carbonate, reflected by the X-ray diffraction results in Figure 1. FA used was classified as a kind of low-calcium fly ash, with a similar X-ray result compared with the study of Zhang et al. [22], which also presented a spherical morphology. As for the used ASR, compared with the study of Xu et al. [23], NaCl or CaCl_2_ cannot be detected by X-ray, showing a lower content of chloride. In contrast, the main components of ASR from soda ash companies tested by the Axios PW4400 wavelength dispersive X-Ray fluorescence spectrometer are calcium carbonate, gypsum and magnesium hydrate (Table 1). Such a significant difference in chemical compositions of FA and ASR is believed to have a great impact on the fresh and hardened performance of MOC paste. FA particles also show a disparate morphology from ASR (Figure 2), distributing dispersedly like micro glass beads with diameters from 1 to 20 μm, while ASR particles generally agglomerate in a sedimentary state, exhibiting an irregular shape. These spherical beads are beneficial for the cementitious solid particles to distribute more homogeneously in the MOC system. FA particles are partially hollow, resulting in a relatively low apparent density of 2.42 g/cm^3^, which is conducive to the production of lightweight MOC products. Particle size distributions of FA and ASR powder are displayed in Figure 3. The threshold diameter for 10%, 50% and 90% cumulative percentages, i.e., D10, D50 and D90, are 1.684 μm, 7.942 μm and 24.23 μm for ASR, 1.699 μm, 14.39 μm and 62.91 μm for FA, respectively. The average particle size of ASR (11.00 μm) is slightly smaller than that of FA (14.39 μm).

### 2.2. Specimen Preparation

To form MOC paste, caustic burnt magnesia with 82.53 wt. % active magnesium oxide and magnesium chloride halide with 48.0 wt. % magnesium chloride were adopted in compliance with QB/T 2605 [24]. The proportions of caustic burnt magnesia (3.58 g/cm^3^), magnesium chloride halide (1.56 g/cm^3^) and water (1.00 g/cm^3^) were optimized as 8:1:12 following previous research [8]. To explore the influence of FA on the properties of MOC pastes, four groups were designed incorporating 10%, 20%, 30% and 40% FA by weight, denoted as Groups A1, A2, A3 and A4. Detailed mix proportions are displayed in Table 2. In contrast, a control group, Group O, of plain MOC paste with no FA addition was prepared to compare the property change in fresh and hardened states of the composites. Mixtures were cast into 40 × 40 × 160 mm^3^ prismatic molds for flexural strength evaluation (three specimens for each group), and a total of six broken specimens for each group were used for the compressive strength test [25]. Moreover, three specimens with a size of 25 × 25 × 280 mm^3^ were adopted for free deformation tests in each group [26]. These specimens were wrapped in plastic films for 24 h at room temperature before demolding and thereafter cured under 20 ± 2 °C and 70 ± 5% relative humidity till the ages for testing. Such curing conditions were determined to simulate the local climate and humidity conditions.

### 2.3. Workability

Flowability of fresh MOC mixtures was determined in a standard truncated cone as per GB/T 8077 [27]. Experiment results are recorded in Table 3. The trend is obvious that the addition of FA leads to an evident decrease in flowability at a fresh state. The reduction rates are 12.5%, 22.04%, 29.32% and 37.50% for Groups A1, A2, A3 and A4, respectively. Possible reasons lie in the increase in solid inert powder dosage and the decrease in water in the whole system [28,29], reflected by mix proportions in Table 2. Furthermore, the smaller average particle size of FA particles (14.39 μm) than caustic burnt magnesia powder (20.82 μm) is also responsible for the lower flowability due to higher water demand for wrapping micro solid particles. Relevant research also proved the improvement of flowability brought by FA in MOC [30].

The setting time was experimentally conducted as per GB/T 1346 [31], recorded from the moment that caustic burnt magnesia was added into magnesium chloride solution (*t*_0_). The initial setting time was measured with the assistance of the Vicat apparatus. A specialized steel needle was applied to estimate the setting of the mixture in concern of the depth that the needle went in the mixture cone. This procedure was conducted every 5 min. Herein, the initial setting was defined as the moment that the distance between the pinhead and the bottom of the cone came to 4 ± 1 mm (*t*_1_). Thus, the time difference (*t*_1_–*t*_0_) was obtained as the initial setting time. Afterward, the needle penetration test was continued every 15 min to obtain the final setting state of the mixture. Similarly, the final setting was defined as the moment that the needle could no longer occur subsidence in the mini-cone (*t*_2_), time difference (*t*_2_–*t*_0_) was calculated as the final setting time. Both types of setting times are listed in Table 3. Clearly, the addition of FA significantly delays the setting of MOC pastes. FA would not directly participate in the early hydration of MOC pastes because of its chemical composition [29]. Moreover, the vitreous nature of FA particles hinders the agglomeration of hydration products in the MgO-MgCl_2_-H_2_O system, which contributes to the elongation of the setting. Interestingly, both initial and final setting time decreases as the dosage of FA increases in the mixtures, attributing to the decreased available free water in the fresh mixture. Such a prolonged effect of the setting process was also reported by Chau and Gong, which showed a delay time of 1–4 h [9,30].

### 2.4. Strength

**Figure 4:**
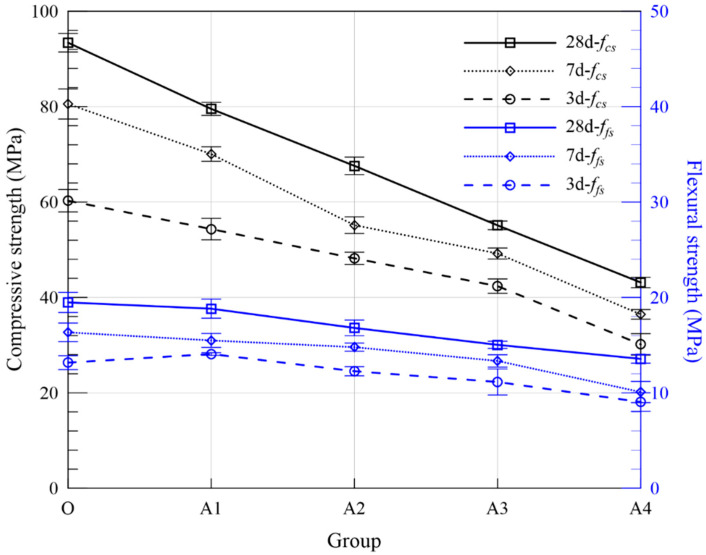
Effect of flexural and compressive strength of MOC-hardened pastes with FA.

Flexural and compressive strength of 40 mm × 40 mm × 160 mm prismatic specimens were conducted according to ISO 679 [25]. Each group contained three specimens from the same batch, resulting in 3 values of flexural strength and 6 values of compressive strength at the ages of 3, 7 and 28 days. The strengths of MOC samples mixed with and without FA are depicted in Figure 4. With the increasing FA dosage, the flexural strength presents a downward trend. Specifically, at the age of 28 days, the flexural strength of Groups A1, A2, A3 and A4 decreased by 0.66 MPa, 2.67 MPa, 4.45 MPa and 5.91 MPa, respectively. The strength reduction in MOC pastes with FA is relatively distinct. As for compressive strength, Groups A1, A2, A3, A4 decreased by 13.87 MPa, 25.80 MPa, 38.28 MPa and 50.26 MPa in comparison with the control group. As an inert additive, a little chemical reaction occurred when FA was added into MOC pastes and the presence of FA prevented the generation of hydration products to some extent [32]. Similar results were obtained by Huang et al., showing the replacement of MgO by FA in MOC will lead to a large strength reduction due to the decreased amount of main hydrate [10]. Moreover, the lubricating effect induced by spherical FA particles would reduce the mechanical interaction with the hydrates, resulting in a negative effect on the strength of hardened pastes [33]. 

### 2.5. Shrinkage

**Figure 5:**
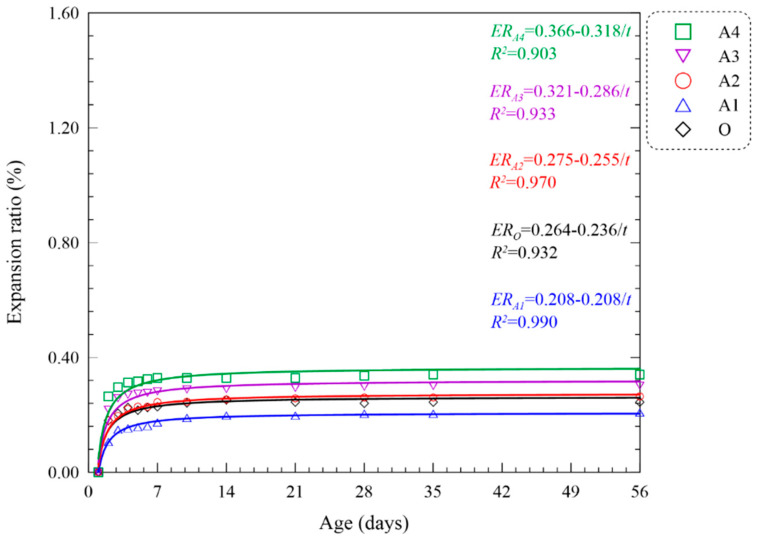
Fitting curve of expansion ratio of MOC pasted with FA.

Free deformation of MOC pastes was measured by strip gauge in compliance with JC/T 313 [26]. The variations were recorded via length gauge throughout the entire curing procedure, indicating the volume stability of slender MOC specimens [8]. Figure 5 displays the expansion ratio of the above five groups from 1 to 56 days. The value of MOC pastes with 10 wt. % FA was maintained at a lower level than plain paste. As active magnesium oxide acts as the expansion source in the MgO-MgCl_2_-H_2_O system, FA plays an important role in delaying the hydration reaction, slowing down the formation of Mg(OH)_2_ crystal and thus inhibiting the expansion of MOC pastes since the early age [34]. Moreover, the acicular 5-phase is transformed to a more stable lamellar 5-phase, stabilizing the volume change of composites. However, as FA further added, a larger expansion ratio was observed by the strip gauge tests, indicating looser microstructure and wider gaps [35]. According to related expansion specifications (0.3% at 28d as per GB/T 33544) [9], the maximum dosage of FA in MOC pastes is limited below 30 wt. %, with an expansion ratio of 0.298% at 28 d.

To further describe the behavior of expansion, hyperbolic fitting is adopted in analyzing the relevance of curing ages and expansion ratio. Fitting curves and equations are displayed in Figure 5. The coefficient of correlation is relatively high (>0.90), implying a reasonable description of expansion ratio development by corresponding equations.

### 2.6. Microstructure Changes

**Figure 6:**
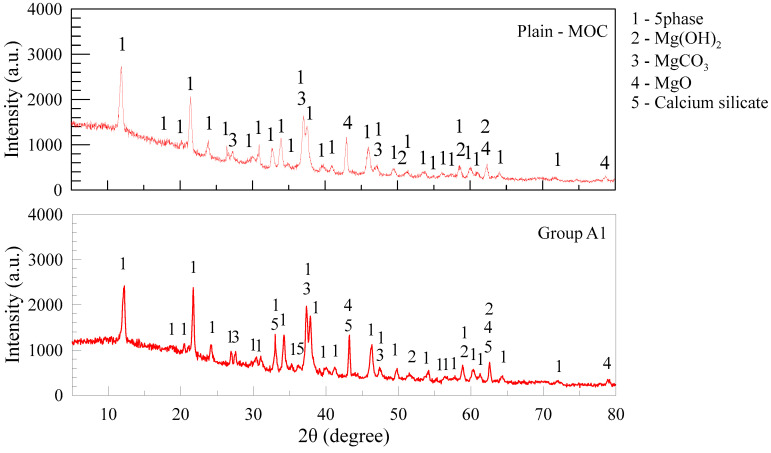
XRD curve of MOC sample.

The samples of MOC pastes were ground to powders with a diameter lower than 0.08 mm for X-ray diffraction (XRD) analyses. Cu is used as the target material, the scan speed is set as 0.017°/min, 2θ in the range of 5° to 80°, and the wavelength is 1.5406 angstrom. Typical XRD results of plain MOC and 10 wt. % FA-MOC are displayed in Figure 6. Mg(OH)_2_, undecomposed MgCO_3_ and unhydrated MgO are found in the plain paste samples together with 5-phase hydrates. Calcium silicate is brought in by FA and no other additional hydration products are observed after mixing FA in the composite. The presence of FA induces a dilution effect and affects the formation of the acicular 5-phase, which is responsible for the strength deterioration as aforementioned.

**Figure 7:**
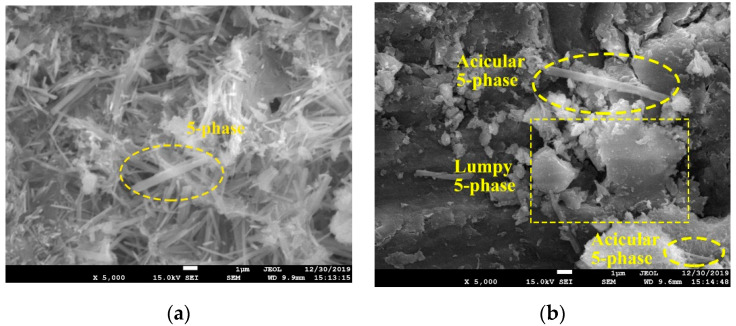

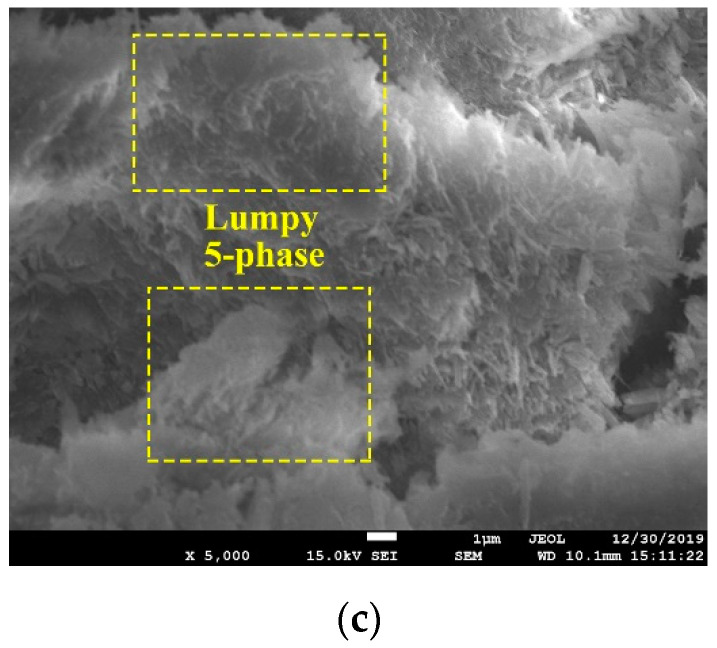
SEM observation of MOC sample (**a**) Group O; (**b**) Group A1; (**c**) Group A4.

For microstructure study under SEM observation, samples were first metal sprayed by Gatan-682 ion etch coating apparatus. Then, the JSM-7001F field emission SEM device with 20 to 200 thousand magnifications was applied for microscope inspections. The resolution is 1.5 nm in low vacuum mode of 10 kV voltage and 1.0 nm in high vacuum mode of 15 kV voltage. From the perspective of micromorphology, the original 5-phase is an acicular structure (Figure 7a), which enlarges the contact surface by intertwining, contributing to the strength formation. Due to the inhibition of FA on the hydration process, the amount of acicular 5-phase becomes less, instead, scattered crystals appear (Figure 7b,c). Such morphology leads to less compactness of the internal structures, thus reducing the strength of MOC pastes [33].

Fly ash particles are characterized by spherical shape like micro glass beads with diameters from 1 μm to 20 μm. The nucleation effect of FA in the magnesium oxychloride cementitious system leads to a clear reduction in flowability but extension of setting. Fly ash inhibits the hydration of active magnesium oxide and brings quartz, calcium silicate and xonotlite into the composite [32]. Consequently, flexural and compressive strength of pastes decrease for all curing ages. However, the reduction of FA on the expansion of MOC pastes is effective, especially when 10 wt. % FA is adopted, which indicates that using FA to improve the volume stability of MOC with industrial waste ASR is technically feasible. To be noticed, the dosage of FA in MOC pastes shall be limited below 30 wt. % according to related expansion specifications [11].

## 3. MOC Pastes with FA and ASR

### 3.1. Mix Proportions and Preparation

**Figure 8:**
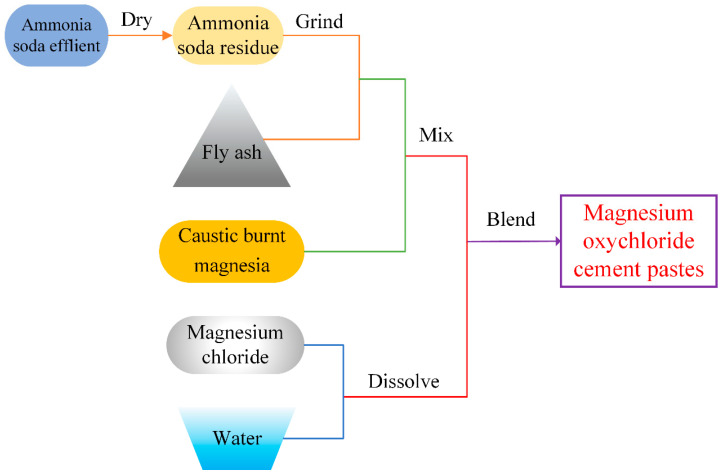
Grinding and mixing processes of ASR and FA.

To evaluate the performance of MOC pastes containing waste ASR, Groups B1, B2, B3 and B4 with 10%, 20%, 30% and 40% ASR by weight were prepared based on previous research [8]. The content of FA in the composite is designed according to the proportion of FA to the total weight of industrial waste, FA/(FA + ASR) = 1:2, 1:1 and 2:1 for Series C, D and E, respectively. Detailed mix proportions are listed in Table 4. Four series in a total of 16 groups with various content of ASR + FA and ratio of FA/ASR are designed to study the combined effects of FA and ASR on MOC pastes. The mixing procedure of the ternary MOC system with both ASR and FA is illustrated in Figure 8. Crude ammonia soda effluent was dried first to a solid state and then ground to ASR powder for blending. Traditional MOC pastes could be obtained by blending caustic burnt magnesia into ready-mixed solutions of MgCl_2_ in a mixing bowl. FA was added simultaneously with ground ASR powder into the composite when necessary. 

### 3.2. Properties of MOC with Industrial Waste

#### 3.2.1. Fresh State Performance

**Figure 9:**
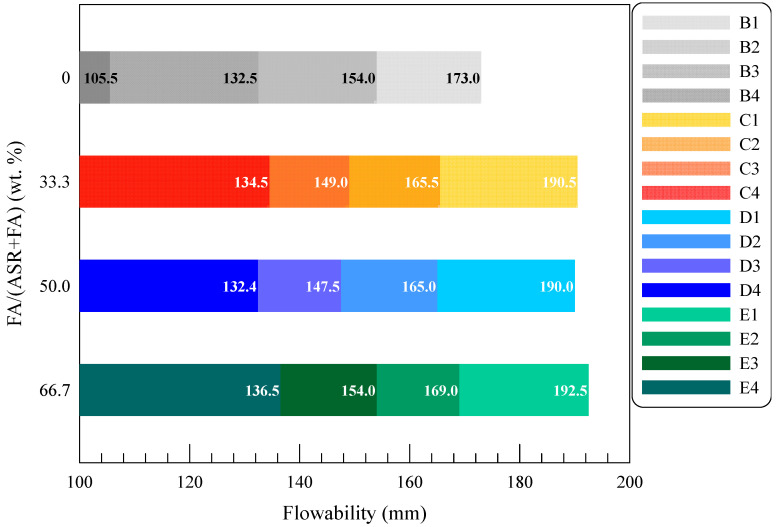
Flowability of MOC pastes with solid wastes.

Flowability of fresh MOC mixtures were determined in a standard truncated cone as per GB/T 8077 [27], and the results of all fresh mixtures are shown in Figure 9. The increasing powder dosage decreases the flowability of MOC pastes. Compared with the control group, the reduction ratios of pastes for Series B lies in the range of 21.36% to 52.05%. The irregular shape and large contact area of ASR powder are responsible for the evident decrease in fluid ability [36]. Nevertheless, when FA is added, the workability of fresh MOC pastes significantly increases in Series C, D and E. In Series C with 33.3 wt. % FA and 66.6 wt. % ASR, the increasing ratios reach 27.49%, 12.45%, 7.47% and 10.12% for Groups C1, C2, C3 and C4, respectively. Such improvements are also observed in Series D and E containing more FA in the system. Better fluid performance displayed in Series C, D and E can be ascribed mainly to the lubricating effect of spherical FA particles [28]. On the condition that the total weight of FA and ASR maintains the same, the increase in FA content leads to higher flowability, benefiting not only the in situ construction but also enhancing the microstructure and durability of the composites.

**Figure 10:**
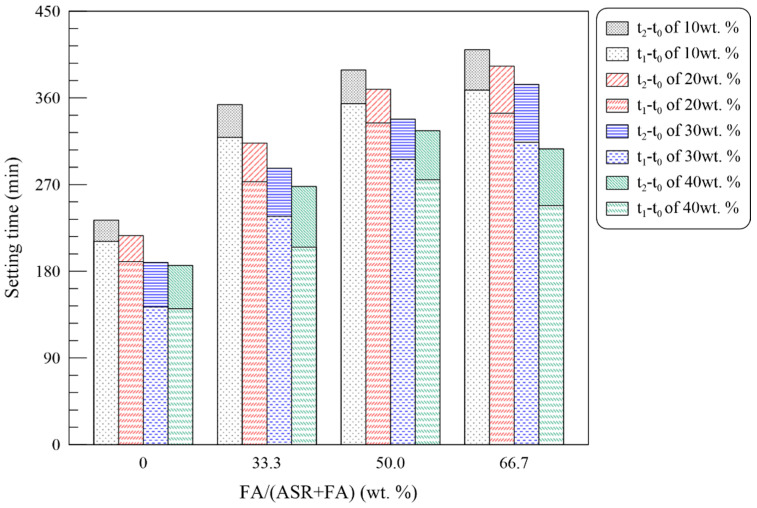
Setting time of MOC pastes with solid wastes.

Setting time was experimentally conducted as per GB/T 1346 [31], and Figure 10 depicts the results from the initial and final setting tests. When ASR is added in Series B, significant decreases in setting time are found. The reduction of free water in fresh pastes and irregular morphologies of ASR particles are two main reasons for the acceleration of the setting process [7]. Furthermore, in the presence of FA particles, the setting process is delayed. For Series C, D and E, both initial and final setting times were prolonged, especially when the dosage of FA is elevated to over 50%. However, the excess admixtures will absorb the free water in the system and provide multiple nuclei in the setting procedure, resulting in a curtailing effect. It should be noticed that the gap between the initial setting and the final setting (*t*_2_–*t*_1_) gets longer when the content of FA becomes higher, which improves the operability of pastes for practical application. 

#### 3.2.2. Flexural and Compressive Strength

**Figure 11:**
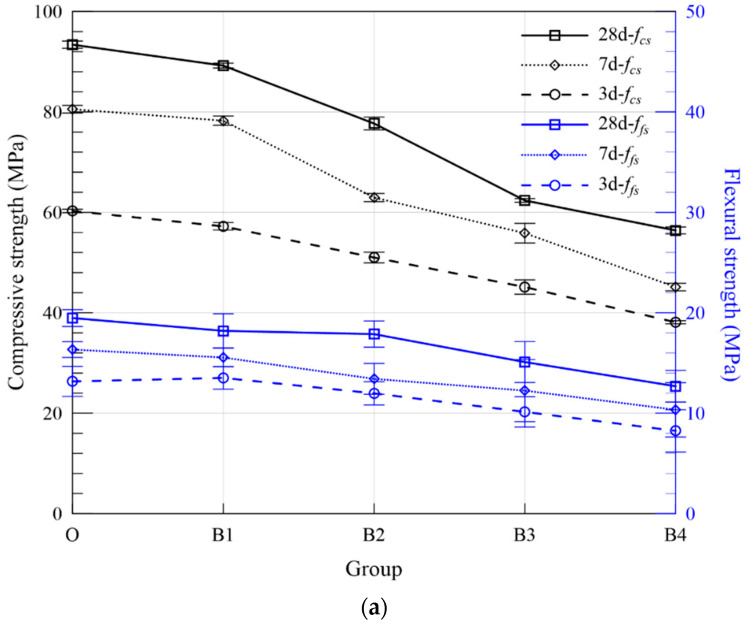

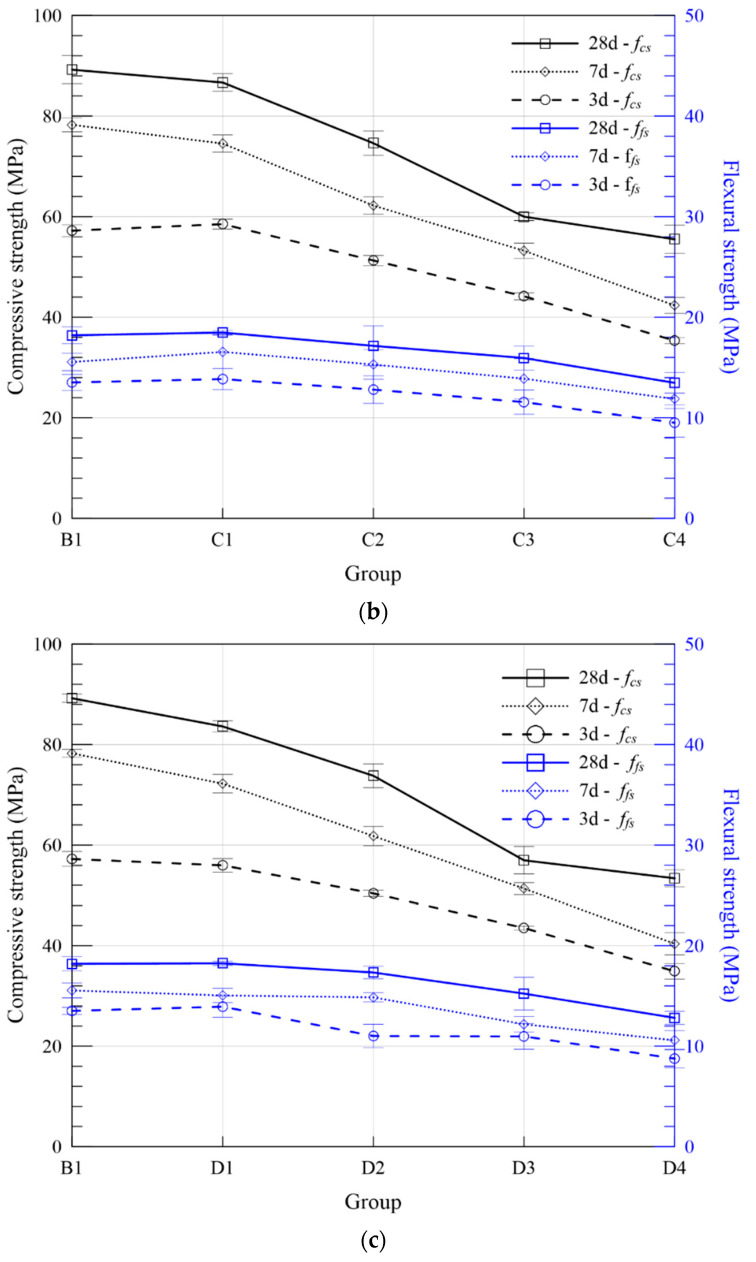

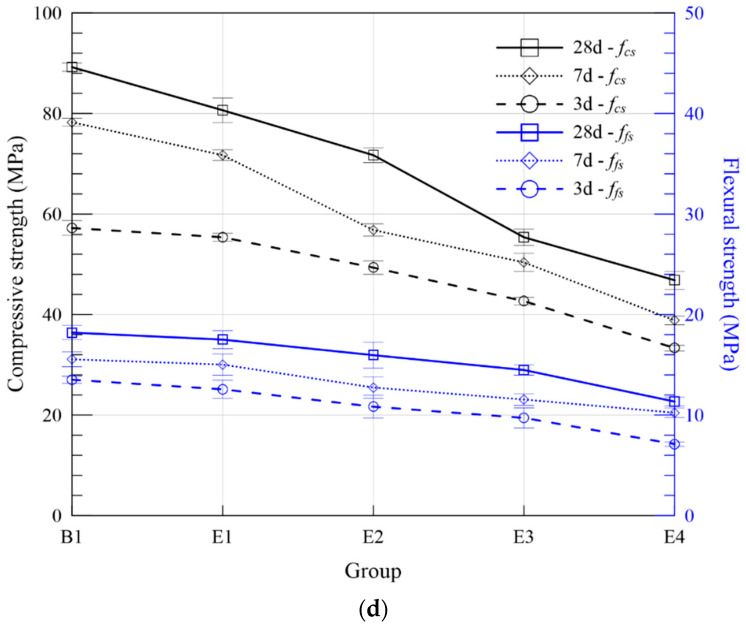
Strength of hardened MOC pastes with FA and ASR (**a**) Series B, (**b**) Series C, (**c**) Series D and (**d**) Series E.

Flexural and compressive strength of 40 mm × 40 mm × 160 mm prismatic specimens were conducted according to ISO 679 [25]. Due to the physical and chemical characteristics of FA particles mentioned above, the strength of MOC pastes incorporating various amounts of FA shall be evaluated. Results are listed in Table 5. On the one hand, by comparing the strength of pastes with the same total weight percentage of ASR and FA, the influence of the FA replacement ratio becomes distinct. For example, when the total ASR + FA is kept constant at 20 wt. %, the 28-day compressive strength for Groups B2, C2, D2 and E2 with 0, 2.51 vol. %, 3.76 vol. %, 5.00 vol. % FA is 77.69 MPa, 74.61 MPa, 73.79 MPa, 71.71 MPa, respectively, showing a monotonic decreasing trend. Such phenomenon is consistent with normal concrete with FA as supplementary cementitious materials, especially at early ages [28]. As for flexural strength, the weakening effect is slightly mitigated, even when the ratio of FA/(FA + ASR) reaches 0.67, the flexural strength of the above four groups after curing for 28 days is 17.88 MPa, 17.16 MPa, 17.34 MPa 15.96 MPa, respectively. On the other hand, Figure 11 depicts the strength variations of MOC pastes with various solid waste percentages for Series B, C, D and E. The compressive strength of MOC paste decreases significantly with the increase in ASR + FA dosage. Take Series D with 50% ASR and 50% FA, for example, the 28-day compressive strength drops from 83.61 MPa to 73.79 MP, 56.95 MPa and 53.37 MPa for Groups D1, D2, D3 and D4, indicating a significant adverse effect of industrial waste on the performance of MOC pastes. The strength difference between Groups D1 and D4 reaches 30.24 MPa, which shall be paid more attention to upon application. Overall, ASR has higher efficiency to compressive strength on MOC than FA, thanks to its irregular shape, which enhances the mechanical interaction. Comparatively speaking, the variation in flexural strength is smaller but also shares a similar descending trend despite the curing ages (3/7/28 days). In general, MOC specimens with 10 wt. % admixture and 33% replacement rate of FA (Group C1) can obtain a higher flexural strength and acceptable compressive strength among all series with composite ASR and FA powder. From the perspective of design and application, the dosage of admixture should be reasonably selected according to the construction demand to avoid large strength reduction.

#### 3.2.3. Volume Stability

**Figure 12:**
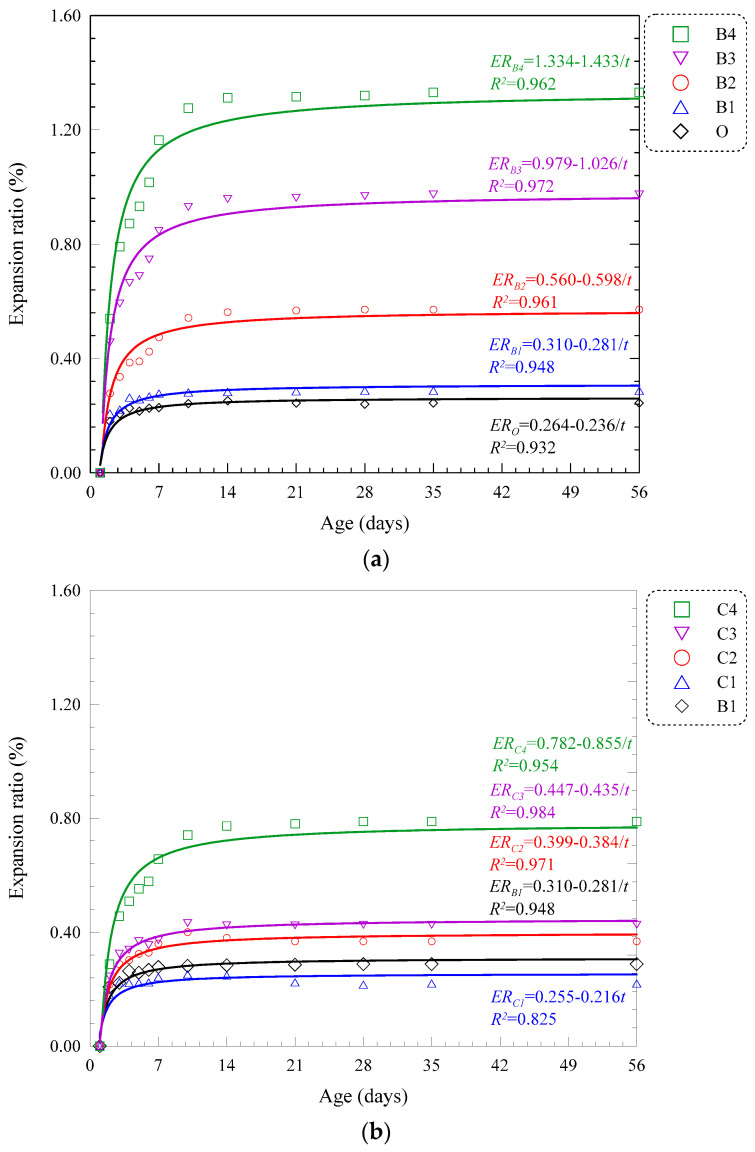

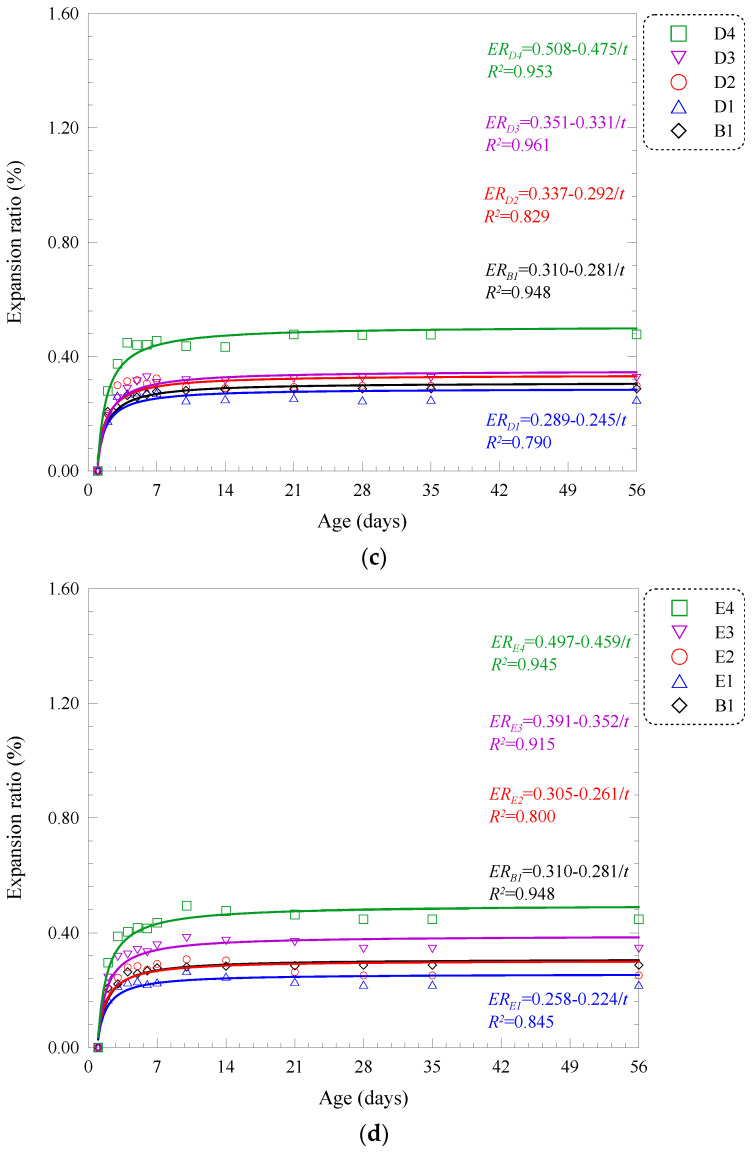
Expansion ratio of blended MOC pastes (**a**) Series B, (**b**) Series C, (**c**) Series D and (**d**) Series E.

Free deformation of MOC pastes was measured by strip gauge in compliance with JC/T 313 [26]. Expansion curves of Series B, C, D and E till 56 days are displayed in Figure 12. As the curing duration extends, all expansion curves exhibit two stages: rapid ascending stage and smooth ascending stage. The first stage generally ends at the age of 7 days, and the deformation developed reaches almost 95% of the total. Such a phenomenon indicates that most of the hydration process is completed in the first 7 days; therefore, the improvement of volume stability of MOC pastes mainly lies in the early age of the composite [37]. Figure 12a shows that the 28-day expansion ratios of MOC pastes with ASR only are 0.288%, 0.571%, 0.973% and 1.331% for B1, B2, B3 and B4, respectively. Higher ASR content leads to higher expansion ratios. When FA is introduced in the composite system, a significant decrease in expansion is observed. Groups C1, C2, C3 and C4 show a 25.00%, 35.55%, 56.00% and 39.94% reduction in terms of ultimate volume change (56 days) in comparison with Groups B1, B2, B3 and B4 with the same total admixture weight. FA exhibits an effective role in mitigating the expansion of ASR-MOC. Such tendency is also found in Groups D1, D2, D3 and D4 with reduction ratio of 13.89%, 47.81%, 66.42% and 63.72%, E1, E2, E3 and E4 of 23.61%, 55.87%, 64.46% and 65.85%. When the proportion of FA/(FA + ASR) increases from 0 to 0.5, the efficiency of FA in expansion mitigation becomes higher, especially when large content of admixture is adopted. However, when the proportion further increases to 0.66, such enhancement is weakened due to inadequate binding materials in the system. Thus, the percentage of FA in the total admixture is suggested to be less than 0.50 for higher mitigation efficiency based on the above results. 

According to the two-stage feature of all expansion curves in MOC pastes, results can be fitted by hyperbolic curves as Equation (1).

(1) ER=m−n/t

where *ER* is the expansion ratio, m and n are two regression parameters and t is the curing age.

The corresponding values of m, n and correlation coefficients are listed in Table 5. Most of the correlation coefficients are larger than 0.94 and the average value of 16 groups is 0.91, indicating a good agreement with a hyperbola. In fact, two parameters are selected for each expansion curve, one related to the ultimate deformation and the other to the ascending rate. Herein, the average expansion ratio of three specimens in Groups C1, D1 and E1 experiences fluctuation compared with other groups, showing data instability when the total volume of ASR and FA is 10%, resulting in a low R2 value. Nevertheless, the overall tendency is consistent.

#### 3.2.4. Microstructure of Blended MOC Pastes

**Figure 13:**
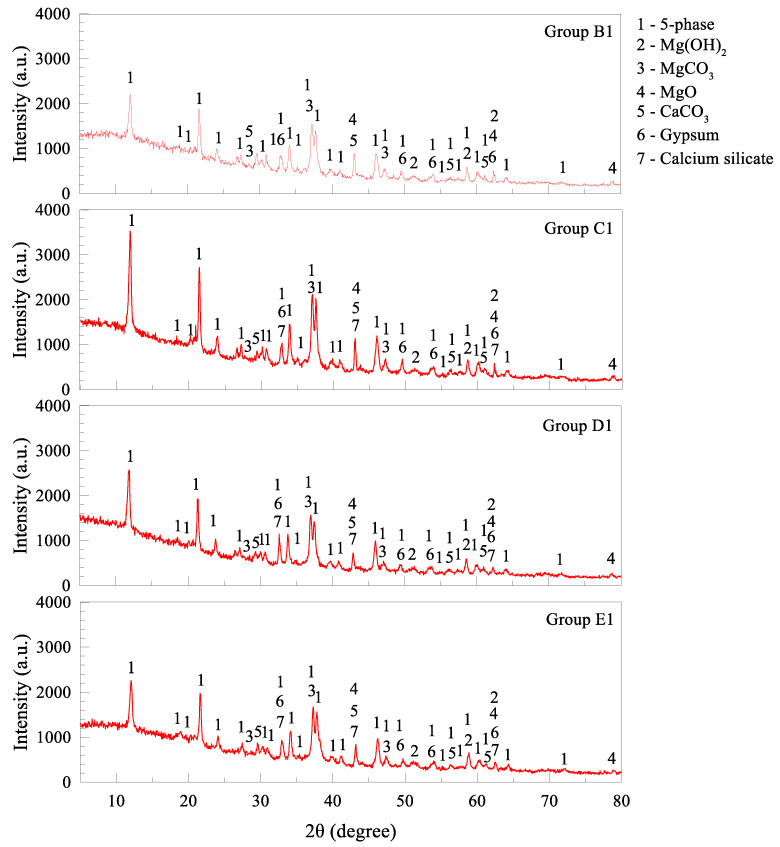
XRD curves of MOC samples with solid wastes.

XRD results of MOC pastes with 10 wt. % (ASR + FA) are displayed in Figure 13, showing the hydration products after curing for 28 days. In comparison with plain MOC paste (Group O), the main difference lies in the presence of CaCO_3_ and gypsum when ASR is incorporated. This is understandable since ASR powder contains a large amount of CaCO_3_ and CaSO_4_·2H_2_O, as listed in Table 1. The main component in all MOC systems is 5-phase (5Mg(OH)_2_·MgCl_2_·8H_2_O), the primary hydration product for strength formation. By comparing XRD results of Groups C1, D1 and E1, the intensity of characteristic peaks differs from each other, indicating different degrees of crystallization. The intensity of 5-phase in pastes with FA is found slightly higher than that with ASR only. As the total content of the admixture increases, the above intensity decreases, which is consistent with the strength reduction mentioned above. Other intensity fluctuations of hydration products are observed in the XRD curves, Mg(OH)_2_ for instance. Figure 6 indicates that when *2θ* is 53.99 degrees, Mg(OH)_2_ crystal is detected, and the intensity is 480 a.u. From Figure 13, this value becomes 328 a.u. at the same *2θ*, indicating a lower expansion rate for Group B1 since Mg(OH)_2_ crystal is the main expansion source for MOC pastes [32]. A similar phenomenon is found in Groups C1 and D1, accounting for the volume stability change. 

**Figure 14:**
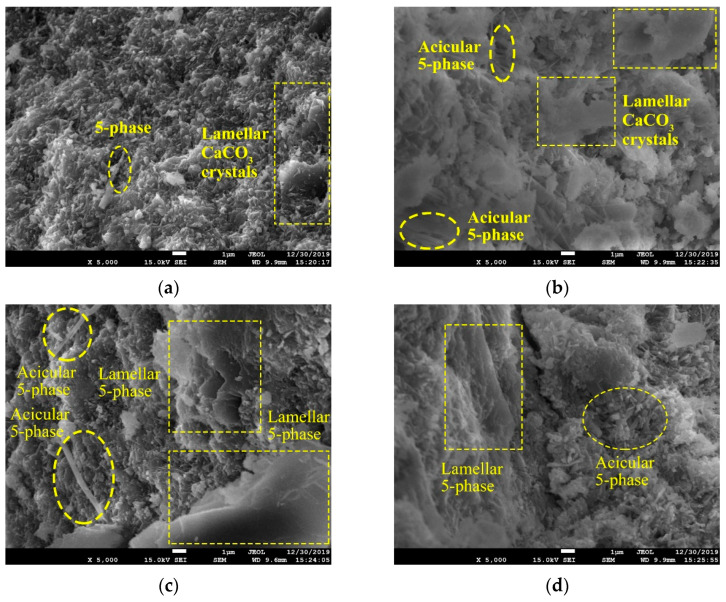

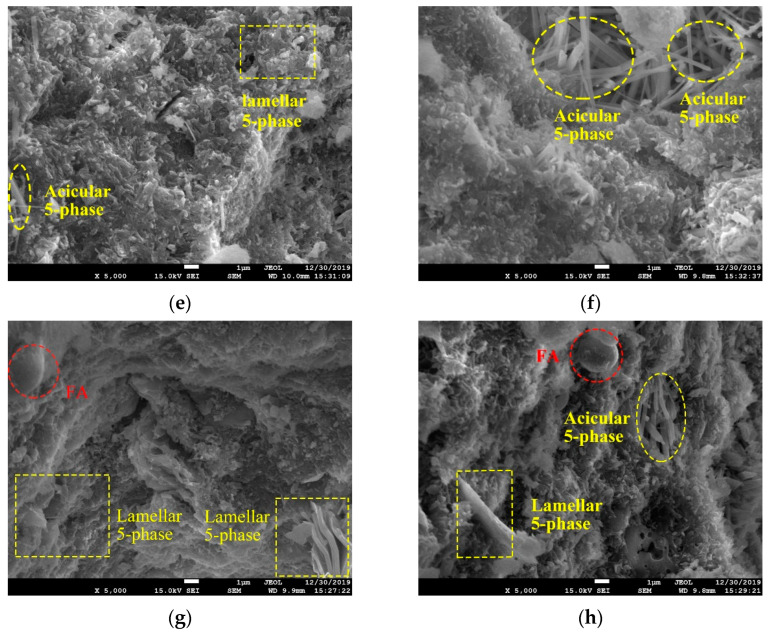
SEM observations of blended MOC samples (**a**) Group B1, (**b**) Group B4, (**c**) Group C1, (**d**) Group C4, (**e**) Group D1, (**f**) Group D4, (**g**) Group E1 and (**h**) Group E4.

Except for the XRD analysis, micro-morphologies of MOC with solid waste ASR and FA are observed and recorded. Figure 14 shows the SEM photos of the MOC sample in Series B, C, D and E. Typical groups with 10 wt. % and 40 wt. % (ASR + FA) are selected for further analysis. As can be seen, the main morphologies of hydrates seldom change even when the addition of industrial wastes reaches 40% by weight. However, the degree of crisscross rate and intertwining effect among crystals descend with the increase in powder content, leading to voids and loose structure in the microscope. This explains the reduction in mechanical properties of hardened cement MOC pastes containing ASR and FA. In Series B, representative acicular 5-phase and lamellar CaCO_3_ crystals are distinct in Figure 14a,b. In comparison, when FA is added, Series C, D and E show less amount of acicular 5-phase but more lamellar and lumpy crystals with different sizes. This can be ascribed to the dilution effect of relatively inert fillers, causing latency in the hydration process. Upon unsaturated situation, 5-phase is difficult to form unstable acicular shape but transforms to stable lamellar and lumpy crystals [38]. Since these crystals are much more stable and would not decompose to Mg(OH)_2_, the volume stability of relevant MOC pastes is improved [39]. Interestingly, no FA particles can be found in Series C and D, but spherical objects are clearly observed in Series E, despite the total percentage of admixtures. Herein, when the proportion of FA to (FA + ASR) reaches 0.66, the composite would exhibit some characteristics of fly ash and show decreased mechanical properties. 

## 4. Analyses and Discussions

### 4.1. Expansion Ratio

**Figure 15:**
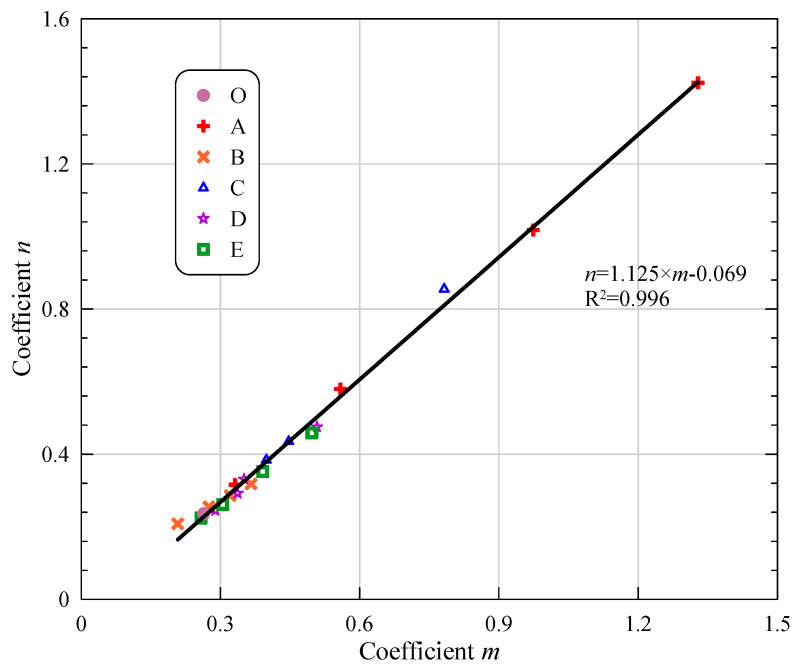
Relationship between coefficient *m* and *n*.

The expansion ratio stands for the length change to the initial length of strip specimens. The experimental results of expansion ratios in all series are shown in Figure 5 and Figure 12. Based on the fitting equations, two parameters are thought to affect the curves of deformation. However, through parameter analysis, a linear relationship between the above two regression parameters m and n can be established with a high correlation coefficient of 0.996, shown in Figure 15 and Equation (2).

(2) $$n=1.125\times m-0.069,\;\;R^{2}=0.996$$

Consequently, the expansion ratio as a function of curing age can be expressed in the form of a single coefficient *a* as Equation (3),

(3) $$ER_{Qi}=a-\left(1.125\times a-0.069\right)/t$$

where *ER_Qi_* is the expansion ratio of Group *Qi* (Q = A, B, C, D, E, *i* = 1, 2, 3, 4), t is the curing age (in the day) and a is a single parameter.

In the early stage (<7 days), the growth rate of the deformation curve is relatively high, which is in line with the rapid ascending of expansion in MOC pastes. As the curing age increases, the value of 1/*t* term becomes smaller, resulting in a gradually stable curve. When *t* goes to infinity, the expansion ratio goes to a, representing the upper limit of the long-term expansion ratio. 

**Figure 16:**
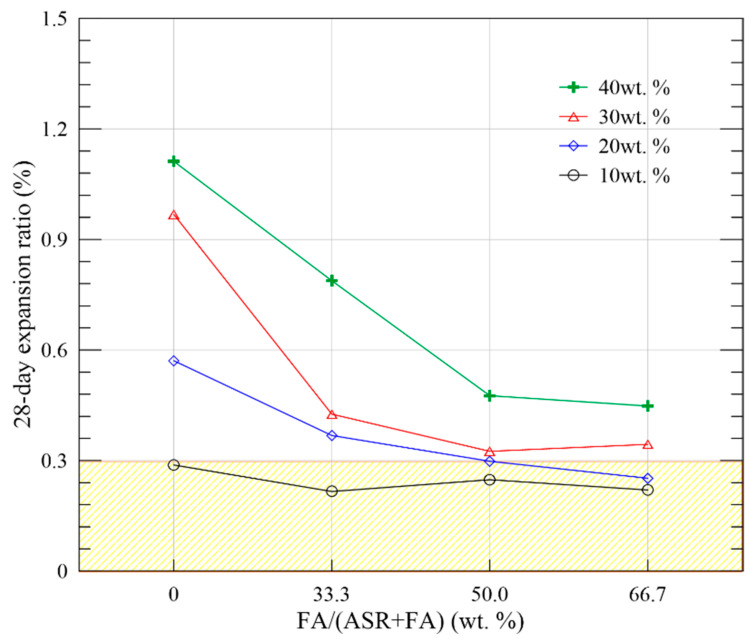
The 28-day expansion ratio of MOC pastes with FA and ASR.

The expansion ratios of Series B, C, D and E at 28 days are depicted in Figure 16. Results show that the deformation ratio of all MOC samples with 10 wt. % admixture meets the specification that below 0.3% in 28 days as per GB/T 33,544 [11]. Moreover, the expansion ratios of Groups D2 and E2 with 20 wt. % (FA + ASR) also meet the above requirement. The incorporation of FA increases the total amount of solid waste in the MOC system, conducive to the recycling of ASR and FA in construction materials.

### 4.2. Simultaneous Expansion-Strength Performance

Deformation tests show that FA can effectively mitigate the high expansion ratio of MOC samples with ASR but induce strength reduction in the meantime. To explore the optimum dosage of ASR and FA, and evaluate the performance of the ternary system compared with the ASR-MOC binary system, a simultaneous strength-shrinkage analysis was put forward. Based on the expansion ratio and compressive strength on 28 days, Equation (4) is proposed below.

(4) $$\beta_{r}=\frac{\left(ER_{Qi}-ER_{Bi}\right)/ER_{Bi}}{\left(f_{cQi}-f_{cBi}\right)/f_{cBi}}$$

where *β_r_* is an index illustrating the rate of relative change in expansion and strength, *ER_Qi_* is the 28-day expansion ratio of Group *Qi* (*Q* = C, D, E, *i* = 1, 2, 3, 4), *ER_Bi_* is the 28-day expansion ratio of Group *Bi* (*i* = 1, 2, 3, 4), *f_cQi_* is the 28-day compressive strength of Group *Qi* (*Q* = C, D, E, *i* = 1, 2, 3, 4) and *f_cBi_* is the 28-day compressive strength of Group *Bi* (*i* = 1, 2, 3, 4).

**Figure 17:**
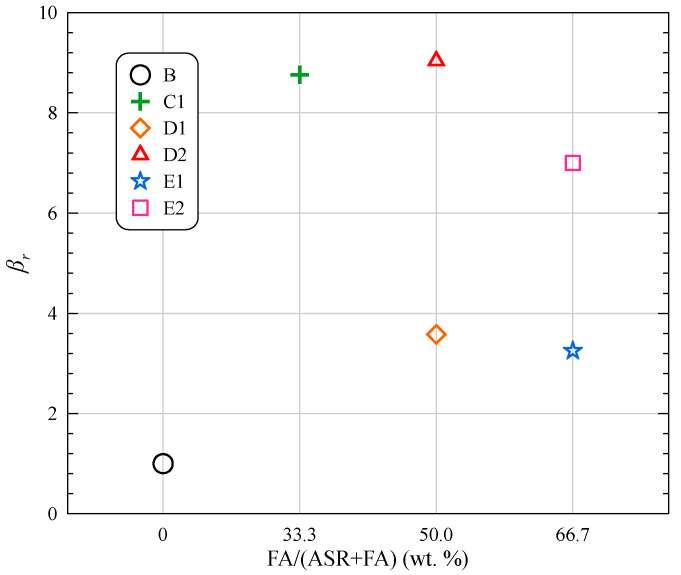
Simultaneous analysis of expansion ratio and compressive strength.

The computed results of all qualified groups (deformation ratio 0.3%) are shown in Figure 17. The value *β_r_* of all groups containing FA is larger than 1.0, indicating a higher improvement in mitigating the deformation of ASR-MOC. When FA is incorporated into ASR-MOC, positive benefits in expansion ratio mitigation are more significant than the negative effect in strength reduction. Through comparison, Group D2 (*β_r_* = 9.04) exhibits the highest expansion-strength performance, consuming up to 20 wt. % industrial waste in the MOC system.

## 5. Conclusions

The present study focuses on the potential recycling of industrial waste FA and ASR in MOC products. Firstly, the effects of FA on the properties of MOC pastes are evaluated. Then, the performance of fresh mixtures containing both FA and ASR were investigated, including flowability, setting time, strength and free deformation. X-ray diffraction (XRD) and scanning electron microscope (SEM) approaches were adopted to illustrate the microstructure changes. Conclusions can be drawn accordingly through analyses and discussions:

1. Recycling solid waste FA and ASR in MOC paste is technically feasible.
2. Inferior flowability and short setting process in the ASR-MOC system caused by irregular ASR can be improved by adding FA.
3. FA would improve the volume stability of MOC, but the maximum dosage is suggested to be lower than 30% by weight.
4. XRD curves indicate that FA affects the intensity of characteristic peaks of 5-phase and Mg(OH)_2_, which well explains the strength and volume stability of ASR-MOC pastes.
5. Through simultaneous expansion-strength performance analysis, up to 20% of solid wastes in weight (10% FA + 10% ASR) can be recycled in MOC products within relevant specifications.

Characterized by great workability, high strength and a low expansion ratio, FA-modified ASR-MOC can be widely used in various construction materials, especially in subgrade materials, high-performance concrete and repair mortar, with long service life. The limitation is also clear that the developed material is not suitable for conditions with high humidity due to its poor water resistance. Fitting equations are proposed according to experimental results, and more complex issues shall be comprehensively considered when applied in constructions.

## Figures and Tables

**Table 1:** Chemical compositions of ASR and FA (wt. %).

Compositions	CaCO_3_	CaSO_4_· 2H_2_O	Mg(OH)_2_	CaCl_2_·2H_2_O	SiO_2_	Al_2_O_3_	Fe_2_O_3_	SO_3_	Others
ASR	56.10	25.50	9.60	3.70	1.60	1.00	0.80	-	0.60
FA	-	-	-	-	57.47	26.56	6.18	0.41	4.01

**Table 2:** Mix proportions of MOC pastes with and without FA.

No.	Series	Groups	FA (wt. %)	FA (vol. %)	MgO (vol. %)	${\mathrm{MgCl}}_{2}$·6H_2_O (vol. %)	H_2_O (vol. %)
1	O	-	0	0	27.34	39.57	33.10
2	A	A1	10	3.89	26.27	38.03	31.81
3		A2	20	7.48	25.29	36.61	30.62
4		A3	30	10.81	24.38	35.30	29.52
5		A4	40	13.92	23.53	34.06	28.49

**Table 3:** Fresh and hardened properties of MOC pastes with and without FA.

Series	Groups	Flowability (mm)	Setting Time (min)		Compressive Strength (MPa)			Flexural Strength (MPa)		Expansion Ratio (%)			Fitting Curve	R^2^
			*t*_1_–*t*_0_	*t*_2_–*t*_0_	*f_c_*_,3_	*f_c_*_,7_	*f_c_*_,28_	*f_f_*_,3_	*f_f_*_,7_	*f_f_*_,28_	28d	56d		
O	-	220	265	299	60.28	80.55	93.39	13.16	16.34	19.48	0.244	0.244	*ER_O_* = 0.264 − 0.236/*t*	0.932
A	A1	192.5	435	479	54.33	70.06	79.52	14.07	15.49	18.82	0.204	0.208	*ER*_A1_ = 0.208 − 0.208/*t*	0.990
	A2	171.5	421	474	48.20	55.16	67.59	12.27	14.79	16.80	0.260	0.264	*ER*_A2_ = 0.275 − 0.255/*t*	0.970
	A3	155.5	403	458	42.37	49.22	55.11	11.14	13.34	15.03	0.298	0.302	*ER*_A3_ = 0.321 − 0.286/*t*	0.933
	A4	137.5	376	446	30.16	36.45	43.13	9.04	10.09	13.56	0.336	0.340	*ER*_A4_ = 0.366 − 0.318/*t*	0.903

**Table 4:** Mix proportions of MOC pastes with composite powder.

No.	Series	Description (in Weight)	Groups	ASR + FA (wt. %)	FA/(FA + ASR)	ASR (vol. %)	FA (vol. %)	MgO (vol. %)	${\mathrm{MgCl}}_{2}$·6H_2_O (vol. %)	H_2_O (vol. %)
1	B	100% ASR + 0% FA	B1	10	0	3.46	0	26.39	38.20	31.95
2			B2	20	0	6.70	0	25.51	36.92	30.88
3			B3	30	0	9.72	0	24.68	35.72	29.88
4			B4	40	0	12.55	0	23.91	34.60	28.94
5	C	66.7%ASR + 33.3%FA	C1	10	0.33	2.31	1.30	26.35	38.14	31.90
6			C2	20	0.33	4.45	2.51	25.43	36.82	30.79
7			C3	30	0.33	6.45	3.64	24.58	35.58	29.76
8			C4	40	0.33	8.32	4.69	23.78	34.42	28.79
9	D	50%ASR + 50%FA	D1	10	0.50	1.73	1.95	26.33	38.11	31.88
10			D2	20	0.50	3.33	3.76	25.40	36.76	30.75
11			D3	30	0.50	4.83	5.44	24.53	35.50	29.70
12			D4	40	0.50	6.23	7.02	23.72	34.33	28.71
13	E	33.3%ASR + 66.7%FA	E1	10	0.67	1.15	2.60	26.31	38.09	31.86
14			E2	20	0.67	2.22	5.00	25.36	36.71	30.71
15			E3	30	0.67	3.21	7.24	24.48	35.43	29.64
16			E4	40	0.67	4.14	9.33	23.65	34.24	28.64

**Table 5:** Properties of hardened MOC pastes with solid wastes.

Series	Group	ASR + FA (wt. %)	Compressive Strength (MPa)			Flexural Strength (MPa)			Expansion Ratio (%)		Fitting Curve	R^2^
			*f_c_*_,3_	*f_c_*_,7_	*f_c_*_,28_	*f_f_*_,3_	*f_f_*_,7_	*f_f_*_,28_	28d	56d		
B	B1	10	57.21	78.26	89.22	13.52	15.55	18.20	0.288	0.288	*ER*_B1_ = 0.310 − 0.281/*t*	0.948
	B2	20	51.00	62.95	77.69	11.98	13.42	17.88	0.571	0.571	*ER*_B2_ = 0.560 − 0.598/*t*	0.961
	B3	30	45.12	55.85	62.37	10.13	12.25	15.09	0.968	0.973	*ER*_B3_ = 0.979 − 1.026/*t*	0.972
	B4	40	38.11	45.12	56.40	8.24	10.34	12.69	1.312	1.331	*ER*_B4_ = 1.334 − 1.433/*t*	0.962
C	C1	10	58.51	74.54	86.67	13.85	16.55	18.48	0.216	0.220	*ER*_C1_ = 0.255 − 0.216/*t*	0.825
	C2	20	51.28	62.21	74.61	12.80	15.30	17.16	0.368	0.368	*ER*_C2_ = 0.399 − 0.384/*t*	0.971
	C3	30	44.17	53.22	59.99	11.56	13.89	15.95	0.426	0.426	*ER*_C3_ = 0.447 − 0.435/*t*	0.984
	C4	40	35.33	42.34	55.51	9.51	11.87	13.48	0.788	0.788	*ER*_C4_ = 0.782 − 0.855/*t*	0.954
D	D1	10	55.95	72.22	83.61	13.94	15.05	18.25	0.248	0.250	*ER*_D1_ = 0.289 − 0.245/*t*	0.790
	D2	20	50.37	61.78	73.79	11.01	14.86	17.34	0.298	0.298	*ER*_D2_ = 0.337 − 0.292/*t*	0.829
	D3	30	43.52	51.34	56.95	10.98	12.19	15.23	0.325	0.325	*ER*_D3_ = 0.351 − 0.331/*t*	0.961
	D4	40	34.91	40.39	53.37	8.77	10.58	12.82	0.476	0.478	*ER*_D4_ = 0.508 − 0.475/*t*	0.953
E	E1	10	55.37	71.71	80.65	12.56	15.02	17.51	0.220	0.220	*ER*_E1_ = 0.258 − 0.224/*t*	0.845
	E2	20	49.34	56.81	71.71	10.83	12.74	15.96	0.252	0.252	*ER*_E2_ = 0.305 − 0.261/*t*	0.800
	E3	30	42.68	50.37	55.37	9.72	11.55	14.48	0.344	0.344	*ER*_E3_ = 0.391 − 0.352/*t*	0.915
	E4	40	33.33	38.83	46.83	7.11	10.23	11.34	0.448	0.448	*ER*_E4_ = 0.497 − 0.459/*t*	0.945

## Data Availability

Data is available upon request.
